# Isolation of Primary Human Hepatocytes from Normal and Diseased Liver Tissue: A One Hundred Liver Experience

**DOI:** 10.1371/journal.pone.0018222

**Published:** 2011-03-29

**Authors:** Ricky H. Bhogal, James Hodson, David C. Bartlett, Christopher J. Weston, Stuart M. Curbishley, Emma Haughton, Kevin T. Williams, Gary M. Reynolds, Phillip N. Newsome, David H. Adams, Simon C. Afford

**Affiliations:** 1 Centre for Liver Research, Institute for Biomedical Research, University of Birmingham, Edgbaston, Birmingham, United Kingdom; 2 Information Technology, University Hospitals Birmingham NHS Foundation Trust, Queen Elizabeth Hospital, Edgbaston, Birmingham, United Kingdom; Chinese University of Hong Kong, China

## Abstract

Successful and consistent isolation of primary human hepatocytes remains a challenge for both cell-based therapeutics/transplantation and laboratory research. Several centres around the world have extensive experience in the isolation of human hepatocytes from non-diseased livers obtained from donor liver surplus to surgical requirement or at hepatic resection for tumours. These livers are an important but limited source of cells for therapy or research. The capacity to isolate cells from diseased liver tissue removed at transplantation would substantially increase availability of cells for research. However no studies comparing the outcome of human hepatocytes isolation from diseased and non-diseased livers presently exist. Here we report our experience isolating human hepatocytes from organ donors, non-diseased resected liver and cirrhotic tissue. We report the cell yields and functional qualities of cells isolated from the different types of liver and demonstrate that a single rigorous protocol allows the routine harvest of good quality primary hepatocytes from the most commonly accessible human liver tissue samples.

## Introduction

High quality primary human hepatocytes are a valuable resource for biomedical research, the pharmaceutical industry and therapeutic purposes [1]–[4]. It is becoming widely accepted that the majority of hepatocyte cell lines lack many important aspects of primary cell function and are unlikely to be used therapeutically. Primary hepatocytes are thus becoming increasingly used in drug development to evaluate key human-specific drug properties such as metabolic fate, drug-drug interactions, and drug toxicity. However the quality and metabolic/functional activity of the cells is variable. Thus human hepatocyte research would be greatly assisted if hepatocytes could be consistently isolated from human liver tissue obtained through hepatobiliary and transplant programs. Previously authors have suggested protocols for successful human hepatocyte isolation but these have applied exclusively to tissue obtained from organ donors [5]–[6] or from non-diseased tissue removed at surgical resection for liver tumours [1], [7]–[10]. Recent work conducted within our laboratory has shown for the first time that human hepatocytes isolated from various cohorts of non-diseased and diseased livers exhibit different responses when used in functional studies [4]. Thus comparative studies need to be carried out in cells isolated from different hepatic diseases.

The isolation of human hepatocytes from steatotic liver tissue has been difficult [6] and although detailed protocols have been described [3] the overall results are so variable in terms of yield and quality of cells that it has been suggested it is not economically viable to attempt hepatocytes isolation from diseased liver tissue [6]. Most published studies have reported outcomes of human hepatocyte isolation from normal resected [1] and donor liver tissue [2], [3]. Here we report our experience of isolating human hepatocytes from all types of livers including normal donor tissue, cut-down liver, normal resected liver tissue and cirrhotic liver disease. We do so in the hope that other researchers in this challenging field may benefit from our experience and make informed decisions on how best to isolate cells for their own studies.

## Methods

### Ethics Statement

Liver tissue was obtained from surgical procedures carried out at the Queen Elizabeth Hospital, Birmingham, UK. Ethical approval for the study was granted by the Local Research Ethics Committee (LREC) (reference number 06/Q702/61). Informed written consent was obtained from all participants involved in the study.

### Human Hepatocyte Isolation

All liver tissue was obtained from fully consenting patients undergoing liver transplantation for a variety of end-stage liver diseases. Human hepatocytes were isolated from explanted diseased livers from patients with alcoholic liver disease (ALD), biliary cirrhosis (primary biliary cirrhosis (PBC) and primary sclerosing cholangitis (PSC) and a variety of other end-stage liver diseases (Cystic Fibrosis, Cryptogenic Fibrosis, Alpha-1-antitypsin Deficiency, Autoimmune Hepatitis and Non-alcoholic Steatohepatitis). Hepatocytes were also isolated from tissue taken from patients who had undergone hepatic resections for liver metastasis from colorectal carcinoma, hepatic resections carried out for benign disease (recurrent cholangitis, focal nodular hyperplasia and haemangiomas), cut-down specimens and normal donor tissue surplus to surgical requirements. For the purposes of the study, liver tissue obtained from resections for benign lesions was included in the normal liver group whereas liver tissue obtained from hepatic resections carried out for metastatic disease was classified as normal resected tissue as is referred to as this hereafter. All the patients included in this latter group had received pre-operative chemotherapy to reduce tumour mass prior to surgery.

For all liver tissue specimens we adopted a stringent and rigorous procurement and isolation protocol. Following explantation or resection, specimens were immediately placed on ice in a sterile sealed draw string bag and processed within 1 hour. There were however exceptions to this, in cases were hepatic resections or liver transplants were carried out at in the late evening or at night, liver tissue was kept sterile on ice overnight. The significant exception to this protocol was in the use of donor liver tissue. In all cases the donor liver was assessed for transplantation by the clinical team at the Queen Elizabeth Hospital, Birmingham and if deemed unsuitable for liver transplantation it was then used for hepatocyte isolation. Precise time between explantation or resection of the liver and commencement of the isolation procedure was recorded in each case.

In isolation procedures where donor liver or liver explants were used, liver wedges were obtained from either the segments II/III or segments V/VI. In procedures where normal resected tissue was used for isolating human hepatocytes, only patients who had undergone right hemihepatectomies were deemed suitable by our pathologist to obtain normal resected liver tissue from. This was obtained in all cases from segments V/VI. Liver wedges were cut and weighed by a trained pathologist. To eliminate interperson variability, the subsequent hepatocyte isolation procedures were carried out by a single individual (R.H.B).

**Figure 1:**
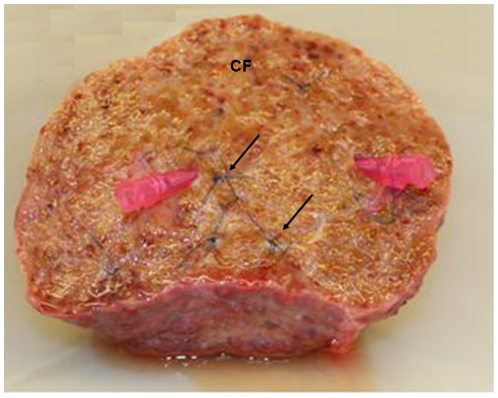
Preparation of the Encapsulated Liver Wedge Prior to Perfusion. Isolation of human hepatocytes was performed from liver wedges (50–413 g). Liver wedges had one cut face (CF) with the remainder of the hepatic capsule being left intact. After washing the liver through exposed vessels with PBS, 20 G cannulae were sutured into suitable vessels with 3/0 prolene purse string sutures. The remaining vessels were oversewn using continuous 3/0 prolene sutures (arrowed). The liver wedge was then ready to be used for the perfusion isolation protocol.

Hepatocyte isolations were carried out using a modified ‘two-stage’ collagenase procedure developed by Berry and Friend [11]. This method has been adapted by several laboratories to isolate human hepatocytes [12]–[14]. After the liver wedge was cut it was placed into ice-cold Dulbecco's Modified Eagles Medium (DMEM) (Gibco, Paisley, UK). The liver was then washed through the exposed, cut, vessels with Phosphate-buffered Saline (PBS), pH 7.2. This was to remove any remaining blood from within the liver wedge and to identify two suitable vessels that could be used for cannulation and subsequent perfusion of buffers. The vessels for cannulation were chosen using two criteria. Firstly, when washing the liver through with PBS it is apparent which parts of the wedge were being ‘perfused’ by that particular vessel, hence the greater the liver area ‘perfused’ the more favourable the vessel was thought to be and secondly the two vessels should be in different parts of the liver wedge to ensure optimum perfusion of the whole wedge. Subsequently two 20 gauge cannulae (Becton-Dickinson, Oxford, UK) were sutured into the chosen vessels using a 3/0 prolene (Covidien, Hampshire, UK) purse string suture (Figure 1). The cannulae were then primed with PBS to ensure that subsequent fluids used for perfusion would run correctly. The final stage of preparing the liver wedge involved oversewing any major vessels that were present on the cut surface of the liver wedge, to ensure minimal loss of perfusion fluids. This also helped maintain pressure within the liver wedge throughout the isolation procedure.

The perfusion circuit was set up according to the widely established routine protocol. One end of the perfusion tube was fed into the perfusion buffer, the central segment of the tubing was within the pump (Model IP 505Du, Watson-Marlow Ltd, Falmouth, UK), and the two outlets of the tubing were connected to the sutured cannulae. The liver wedge was held over a container during the procedure. This container also contained the waste tubing which connected the container to the waste pot. At the commencement of the procedure, all rubber tubing remained ‘open’ allowing the free flow of perfusion and waste fluids. All buffers used in the isolation procedure were pre-warmed to 42°C in a water bath. At this stage, liver wedges were first perfused with ‘non-recirculating’ wash buffer (10 mM 4-(2-hydroxylethyl)-1-piperazineethanesulfonic acid (HEPES) pH 7.2) (Sigma, Dorset, UK) at room temperature using a flow rate of 75 ml/min, ensuring flushing out of blood within the liver vasculature. After this, the wedge was perfused with chelating solution (non-recirculating) (10 mM HEPES, 0.5 mM Ethylene Glycol Tetraacetic Acid (EGTA), pH 7.2) (Sigma) in order to disrupt cell adhesion to the underlying matrix. This was followed by further perfusion with (non-recirculating) wash buffer to remove any remaining EGTA from the liver as the enzymes used to dissociate the liver are dependent upon calcium and magnesium for activation. At this point the waste tubing was clamped to allow recirculation of the enzymatic buffer. Enzyme buffer perfusion solution was made up as follows: Fresh aliquots of enzymes were removed from −20°C storage and dissolved in Hank’s Balanced Salt Solution (HBSS) (Gibco, Paisley, UK) that had been supplemented with calcium chloride (5 mM) and magnesium chloride (5 mM). Following enzyme dissolution the solution was filtered through a sterile filter (Miltenyi Biotec Ltd, Surrey, UK) back into the HBSS solution. Specifically, 0.5% w/v Collagenase A (from Clostridium Histolyticum, Roche, Hertford, UK, Lot number 70273822), 0.25% w/v Protease (Type XIV from Streptomyces Griseus, Sigma, Lot number 076K1177, 4.5 units/mg), 0.125% w/v Hyaluronidase (from bovine testes, Sigma, Lot number 025K7015, 451 units/mg) and 0.05% w/v Deoxyribonuclease (from bovine pancreas, Sigma, Lot number 107K7013, 552 units/mg) were the enzymes used. The liver wedge was perfused with the recirculating enzyme solution at 37°C using a flow rate 75 ml/min for between 1–19 min, this time was designated to be the perfusion time. The decision to stop perfusion was made when the cut surface of the liver allowed the admission of a digit. At this point the perfusion tubing and cannulae were removed and the liver was then transferred to a sterile glass dish and dissociated using manual force whilst in DMEM supplemented with 10% v/v heat inactivated foetal calf serum (Gibco), 2 mM glutamine (Gibco), 20,000 units/l Penicillin, and 20 mg/ml Streptomycin (Gibco) and 2.5 µg/ml Gentamycin. Following manual dissociation of the liver wedge the suspension was passed through a sterile nylon mesh of 250 µm (John Staniar Ltd, Manchester, UK) followed by a sterile nylon mesh of 60 µm (John Staniar Ltd). Suspensions were then washed three times at 50×g for 10 min at 4°C in supplemented media.

**Figure 2:**
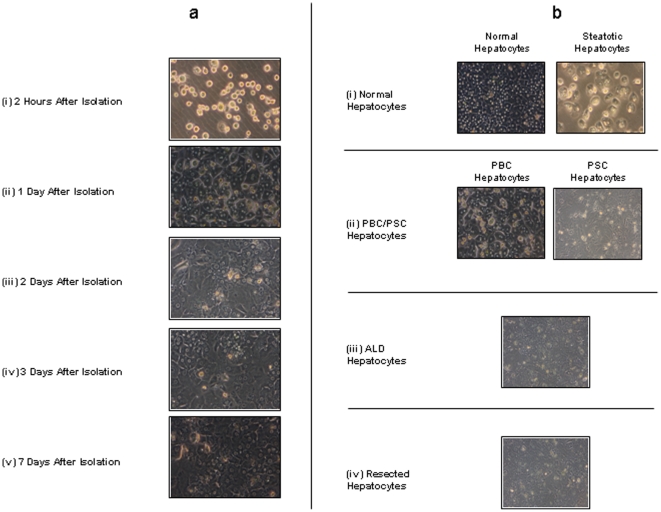
Morphology of Primary Human Hepatocytes after Isolation in Culture. (a) Comparison of the morphological appearance of primary human hepatocytes isolated from primary biliary cirrhosis (PBC) attached to collagen-coated plates at (i) 2 hours after isolation (magnification 20×), (ii) 1 day after isolation, (iii) 2 days after isolation, (iv) 3 days after isolation and (v) 7 days after isolation using light microscopy. The representative images show the change in morphology of primary human hepatocytes isolated from PBC livers during 1-week in culture. Immediately after isolation, cells appear ovoid and phase bright. Following 1 day in culture the cells appear binucleate and have formed a confluent monolayer. Primary human hepatocytes maintain this morphology throughout the one week culture period. Similar morphological changes were observed for human hepatocytes isolated from normal, ALD and normal resected liver tissue (data not shown). (b) Representative images of the morphology of human hepatocytes isolated from normal, PBC/PSC, ALD and normal resected liver tissue are shown in culture 3 days after successful cell isolation. Human hepatocytes demonstrated and maintained this morphology for at least one week following successful isolation. (i) Demonstrates the morphology of human hepatocytes isolated from normal liver tissue and macro-steototic liver tissue (magnification 20×). Primary human hepatocytes isolated from normal liver tissue display the typical cubic binucleate cell morphology. In contrast, primary human hepatocytes isolated from overtly macro-steatotic livers demonstrate obvious micro-vesicular steotosis. (ii) Human hepatocytes isolated from end stage biliary cirrhosis (PBC/PSC) again show the typical features of cells in culture with a cubic and binucleate morphology. (iii–iv) Cells isolated from ALD and normal resected liver show similar morphological features to human hepatocytes isolated from normal non-steatotic livers and biliary cirrhosis but also exhibit micro-vesicular steatosis.

**Figure 3:**
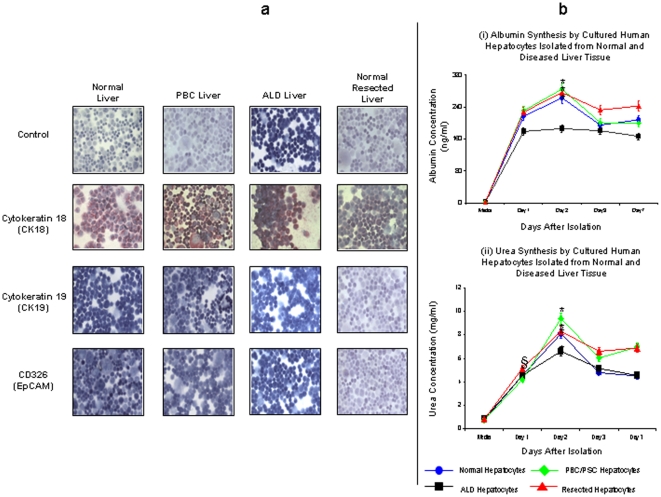
Immunostaining of Cytokeratin 18, Cytokeratin 19 and CD326 and Metabolic Activity of Primary Human Hepatocytes Isolated from Normal and Diseased Liver Tissue. (a) Human hepatocytes isolated from normal, PBC, ALD and normal resected liver tissue show strong staining for the intracellular hepatocyte marker Cytokeratin 18 (CK18). In contrast, isotype matched controls immunoglobulin showed no positivity. Furthermore, isolated human hepatocytes showed no staining with the biliary epithelial cell marker Cytokeratin 19 (CK19) or hepatic progenitor cell marker CD326 (EpCAM). Cytospins of human hepatocytes were made immediately after successful cell isolation, fixed in acetone for 5 min and stored at −20°C until immuno-staining was performed. (b) Following successful isolation, human hepatocytes isolated from normal, PBC/PSC, ALD or normal resected liver tissue were able to synthesise albumin (i) for at least one week demonstrating active metabolic activity in culture. Human hepatocytes isolated from normal, PBC/PSC and normal resected liver tissue show significantly greater albumin synthesis after two days in culture (*p<0.05). Morover, human hepatocytes isolated from ALD livers synthesised significantly less albumin than other types of human hepatocytes (p<0.05). In addition, human hepatocytes isolated from normal resected liver tissue synthesised significantly greater amounts of albumin than normal hepatocytes (p<0.037). (n = 3–4 separate samples). Human hepatocytes isolated from normal, PBC/PSC, ALD and normal resected liver tissue were also able to synthesise urea (ii) for at least one week following successful cell isolation. Human hepatocytes isolated from both normal and diseased liver tissue synthesised significantly lower levels of urea one day after isolation (§p<0.001) compared to the remainder of the culture period, but all human hepatocytes produced significantly more urea on day 2 when compared to other days (*p<0.001). Finally, PBC/PSC and normal resected human hepatocyte synthesis significantly greater amounts of urea when compared to the other human hepatocytes (p<0.05) but there was no significant difference between PBC/PSC and resected human hepatocytes. (n = 3–4 separate samples).

Immediately after washing, cell viability was determined by trypan blue dye exclusion and deemed acceptable if greater than >50% at this stage. Hepatocytes were then plated out in supplemented media. If viability was lower but total cell yield was high, Percoll density gradient centrifugation of cell suspensions was carried out to improve yield of viable cells. Percoll (Sigma) was made by adding to PBS pH 7.2 to density of 4.5 g/ml. Percoll was added to cell suspensions and washed at 300×*g* for 30 min at room temperature. Cell viability was again determined by trypan blue dye exclusion and if improved to >50% cells were plated out at 5×10^5^ per well in supplemented media for 2 hours onto type 1 rat collagen coated plates to allow adherence of cells. After this period, the media was changed to Arginine-free Williams E media (Sigma) containing Hydrocortisone (2 ug/ml), Insulin (0.124 U/ml), Glutamine (2 mM), Penicillin (20,000 units/l), Streptomycin (20 mg/l) and Ornithine (0.4 mmol/l). The media was exchanged every 24 hours and for subsequent functional studies, cells were used within 2 days. Figure 2 shows the morphology of primary human hepatocyte isolated from PBC livers at different time points following successful isolation. The morphology of human hepatocytes isolated from normal tissue, biliary cirrhosis, ALD and normal resected liver tissue three days after successful isolation are also demonstrated (Figure 2b). As Figure 3 demonstrates, primary human hepatocytes showed characteristic phenotypic features of hepatocytes and were able to maintain urea and albumin synthesis up to at least one week following successful isolation. Successful human hepatocyte isolations were defined as successful plating of cells that were used in subsequent functional studies.

Liver samples from the normal liver category were histopathogically evaluated on formalin fixed paraffin-embedded sections by G.M.R. Steatosis was reported by the percentage of steatotic hepatocytes present and group accordingly: minimal/none = 0–5%, mild = 5–33%, moderate = 33–66% and severe = >66%.

### Urea Synthesis Assay

Confirmation of urea synthesis by isolated human hepatocytes was performed using quantitative colorimetric urea determination (QuantiChrom™ urea assay kit-DIUR-500) (Bioassay Systems, Hayward, CA, USA).

### Albumin Synthesis Assay

Albumin synthesis by isolated human hepatocytes was confirmed using a sandwich ELISA kit (Abnova, CA, USA).

### Immunostaining human hepatocytes for Cytokeratin 18

Following successful isolations, cytospins of hepatocytes were made on Poly-L-lysine coated glass slides and fixed for 5 min in acetone. Cells were then incubated with 20% normal rabbit serum (Dako, Cambridge, UK) in Tris-buffered saline pH 7.4 (TBS) for 30 min. Monoclonal antibody to Cytokeratin 18 diluted 1∶100 (clone DC10, Dako) or monoclonal antibody to Cytokeratin 19 diluted 1∶100 (clone RCK108, Dako) or monoclonal antibody to CD326 (Epithelial Cell Adhesion Molecule (EpCAM) diluted 1∶100 (clone HEA125, Progen, Heidelberg, Germany) was applied for 60 min, followed by polyclonal rabbit-anti mouse IgG antibody (1∶25 dilution, Dako) for 30 min and finally anti-alkaline phosphatise (APAAP) complex (1∶50 dilution in TBS) for 30 min. After each incubation step cytospins were washed x3 in TBS. Antibody binding was visualised with 0.2 mg/ml naphthol-AS-MX-phosphate dissolved in Dimethylforamide, (Sigma), 1 mg/ml Fast Red (Sigma) in 0.1 M Tris buffer (pH 8.2) and 0.25 mg/ml Levamisole (Sigma) for 15 min followed by counterstaining with Mayer's haematoxylin (Dako). Control cytospins were included where primary antibody was substituted for isotype matched immunglobulin. Slides were then mounted with Immunoblot mounting medium (Dako) and assessed for positivity.

### Statistical Analysis

Univariable analysis was performed to test the effect of specific factors upon outcome measures, human hepatocyte cell viability following a preparation, total cell count after preparation and success. Success was defined as maintenance of cell adherence and morphological integrity 48 hours after plating onto type I rat collagen and their subsequent use in functional studies. The specific factors we analysed were hepatic disease type, time delay between hepatectomy and beginning of the liver perfusion, weight of liver wedge, perfusion time, the level of steatosis in the normal liver cohort, and the use of Percoll enrichment. In univariable analysis, Mann-Whitney tests were used for the time and weight variables, as they did not to follow a normal distribution, with Fisher's Exact test being used for the categorical variables. Multivariable analysis was performed by considering all specific factors simultaneously and assessing the effect upon the said outcome measures. For this analysis logistic regression models were used.

Repeated-measures ANOVA was utilised to analyse the synthesis of albumin and urea by human hepatocytes isolated from normal, PBC/PSC, ALD and normal resected liver tissue.

## Results

Liver wedges were prepared as shown in Figure 1 and processed as detailed in the Methods section. Figure 2a shows representative images of primary human hepatocytes, isolated from a liver with PBC, in culture for the first week after isolation. Similar morphological changes were observed during the first week after successful cell isolation from normal, PSC, ALD and normal resected liver tissue. Figure 2b demonstrates the morphological features of primary human hepatocytes isolated from these various livers 3 days after isolation. The morphology of primary human hepatocytes after 3 days in culture was maintained for at least one week after successful isolation.

The type of livers used and the main results of all human hepatocyte isolations are shown in table 1. We processed and isolated human hepatocytes from 104 liver wedges over a two year period. A variety of liver diseases were used including, as detailed above, ALD, biliary cirrhosis (PBC and PSC), normal resected liver and normal liver tissue (donor liver tissue, resections for benign liver disease and cut-down specimens). A large proportion of livers used in our study were from cirrhotic, end stage liver diseases (54%). Normal resected liver tissue was defined as liver tissue from patients with colorectal metastasis accounted for 26% of livers used. All these latter patients had received pre-operative chemotherapy for reduction of tumour mass prior to surgery.

**Table 1:** The Type of Livers used and the Main Results of All Human Hepatocyte Isolations.

Liver Type	Time Delay(hrs)	Weight(g)	Perfusion Time(min)	Absolute Cell Count After Perfusion×10^3^	Viability(%)	Success Rate(%)
Normal (n = 21)	2.5(1–14)	110(66–413)	1.5(1–11)	57(75–200,000)	46(0–100)	53
Donor Liver (n = 7)	4(2–14)	117(101–150)	1.5(1–11)	1500(75–200,000)	20(0–60)	29
Normal Liver (n = 14)	2(1–4)	105(66–413)	2(1–7)	505(75–32,000)	50(10–100)	64
Resected (n = 27)	2.5(1–16)	90(50–180)	1.5(1–5)	220(50–6,000)	50(0–100)	53
Biliary Cirrhosis (n = 24)	2(1–11)	106(78–200)	1.5(1–8.5)	720(200–7,000)	55(0–100)	71*
ALD (n = 24)	2.5(1–13)	110(64–220)	3(1–19)	155†(20–3,000)	40(0–100)	29
Others (n = 8)	4.5(1–12)	101(56–115)	5.5(3–17)	225(40–3,000)	7.5(0–70)	13
TOTAL (104)	2(1–16)	110(50–413)	2.5(1–19)	350(20–200,000)	40(0–100)	51

The time delay, weight of liver wedges, perfusion time, absolute cell count after perfusion, cell viability and success rate are shown for various liver categories; Normal (donor liver tissue, normal benign tissue and cut-down specimens), normal resected liver tissue, biliary tissue (PBC and PSC), ALD and others (cystic fibrosis 2, cryptogenic fibrosis 2, alpha-1-antitypsin deficiency 1, autoimmune hepatitis 1 and non-alcoholic steatohepatitis 2). All values are represented as medians and the values in parentheses represent the range. n, number of cases.

† p<0.05, multivariable analysis showing ALD livers yield significantly lower cell yields when compared to other liver diseases.

*p<0.05, multivariable analysis showing biliary cirrhosis yielded a higher success rate of human hepatocyte isolation when compared to other liver diseases.

Isolated cells from human liver tissue were all positive for the hepatocyte cell marker, Cytokeratin 18 and were negative for the biliary epithelial cell marker, Cytokeratin 19, and the hepatic progenitor cell marker, CD326 (EpCAM) (Figure 3a). Furthermore, all hepatocytes isolated from normal and diseased liver tissue demonstrated albumin and urea synthesis in the first week after isolation (Figure 3b). Human hepatocytes isolated from normal, normal resected and biliary cirrhosis showed significantly increased albumin synthesis after two days in culture. Interestingly, human hepatocytes isolated from ALD livers demonstrated significantly reduced levels of albumin production when compared to other hepatocytes (p<0.05). Urea synthesis by human hepatocytes demonstrated similar characteristics, with production of urea being significantly greater on day 2 when compared to other days in culture. Of note biliary cirrhosis and normal resected human hepatocyte produce significantly more urea in culture when compared to other human hepatocytes.

Absolute cell count after isolation was an important parameter on which to assess efficacy of the procedure. Univariable analysis revealed that disease type (p<0.001, Fisher's Exact test) was the only factor to have a significant bearing upon total cell count after primary isolation. Multivariable analysis showed that ALD livers have a significantly lower cell yield when compared to other liver categories (p<0.15 – table 1).

Overall, for all human hepatocyte isolations we report a median cell viability of 40%. Human hepatocytes isolated from biliary cirrhosis (PBC and PSC) had the highest median cell viability (55%). Univariable and Multivariable analysis showed the only factor which had an effect upon improving cell viability was the time delay between hepatectomy and beginning of the perfusion procedure (p<0.05, Mann-Whitney test). Complete processing within 3 hours resulted in a significantly higher cell viability (>50%), compared to time delays greater than 5 hours (p<0.05, Odds Ratio  = 3.594). Whether the delay is 3–5 hours or more than 5 hours did not have a significant effect on the likelihood of the cell viability being greater than 50% (p = 0.375, Odds Ratio  = 1.725). These data are summarised in Table 2. In our study we used Percoll density gradient centrifugation to enrich hepatocytes purity where cell viability was low but absolute cell count high (n = 8). These data are summarised in Table 3. Percoll enhanced purity of human hepatocyte isolates but at the expense of cell count, hence the need for a relatively high absolute cell count after the initial liver preparation.

**Table 2:** Effect of Time Delay Between Hepatectomy and Beginning of the Perfusion Procedure Upon Human Hepatocyte Cell Viability Following Isolation Procedure.

Time Delay (hrs)	Median Cell Viability (%)
<3	53
3–5	25
>5	20

**Table 3:** Effect of Percoll upon Cell Viability and Absolute Cell Count.

Absolute Cell Count after Preparation×10^3^	Viability(%)	Absolute Cell Count after Percoll×10^3^	Viability after Percoll(%)
550(200–200,000)	35(10–50)	175(100–400)	85(55–90)

The effects of Percoll upon human hepatocyte isolation procedures. We used Percoll after eight human hepatocyte isolation procedures (normal resected liver tissue 1, biliary cirrhosis 5, donor liver tissue 1 and normal benign liver tissue 1). All values are represented as medians and the values in parentheses represent the range.

We assigned all donor liver tissue, resections performed for benign liver disease (recurrent cholangitis, focal nodular hyperplasia and haemangiomas) and cut down specimens as ‘normal’ liver tissue. These livers had no signs of intrinsic liver disease. This group was analysed as a whole and as a sub-group, where donor liver tissue was compared to normal liver tissue (resections from benign liver disease and cut down specimens). Sub-group analysis was carried out to determine whether the level of steatosis had an effect upon absolute cell count, cell viability and success rate of subsequent culture. The level of steatosis in the two groups is shown in Table 4. We found no difference in absolute cell count, cell viability, and success rate between the donor liver and normal liver groups. The success of isolating human hepatocytes from this group as a whole was good (53%) but normal liver tissue obtained from hepatic resections carried out for benign disease and cut-down specimens had a statistically higher success rate (64%) when compared to donor liver tissue (20%).

**Table 4:** The Level of Steatosis in Liver Wedges taken from Donor Liver Tissue and Normal Liver Tissue.

	Minimal/None	Mild	Moderate	Severe
Donor liver tissue (n = 6)	1	1	3	1
Normal liver tissue (n = 12)	10	1	1	-

The level of steatosis in liver wedges taken from normal liver tissue. The level of steatosis was classified as minimal/none = 0–5%, mild = 5–33%, moderate = 33–66% and severe = ≥66% after histological analysis of paraffin embedded sections. We were unable to classify the level of steatosis in three liver wedges (1 donor liver and 2 normal livers) as the tissue from those particular livers was not available. n, number of cases.

Our series is the largest reported of human hepatocyte isolations from cirrhotic livers (n = 55). We report a median success rate when isolating hepatocytes from cirrhotic livers of 45%. Table 1 shows that liver wedges from biliary cirrhosis (PBC and PSC) had the highest success rate of all livers used in our study (71%). For all liver isolations, univariate analysis showed a significant difference in time delay between hepatectomy and beginning of liver perfusion (p<0.001, Mann-Whitney test), perfusion time (p<0.05, Mann-Whitney test) and disease type (p<0.05, Fisher's Exact test) between the groups of successful and unsuccessful isolations. Multivariable analysis revealed disease type (p<0.05) and time delay between hepatectomy and beginning of perfusion (p<0.05) were significant factors affecting successful human hepatocyte isolation. As described above for cell viability, success is significantly dependent upon time delay (p<0.01), with a delay of less than 3 hours resulting in improved success rates compared to those of more than 5 hours (p<0.05, Odds Ratio  = 3.735).

## Discussion

The preparation of primary human hepatocytes is a time consuming, logistically demanding, and expensive process. The demand for high quality human hepatocytes for cell transplantation and pharmo-toxicological studies continues to rise. However, the availability of human liver tissue for laboratory investigation remains limited. Therefore, cirrhotic end-stage livers, which are more readily available to researchers, represent another potential source of human hepatocytes. We know of no other studies which evaluate and compare the outcome of human hepatocytes isolation from all sources, including end-stage liver disease, using one standard isolation protocol. Our experience highlights that the isolation of primary human hepatocytes from normal or diseased livers is a viable proposition given appropriate methods.

Our data confirm that isolating human hepatocytes from ALD livers is technically challenging and we had a success rate of only 29%. Thus the use of ALD livers for hepatocyte isolation is a questionable proposition. This may in part be explained by the variable disease stage of our ALD cohort which included patients with advanced cirrhosis or it may reflect toxicological damage resulting from the effects of alcohol. Excluding the ‘other’ disease categories, ALD livers had the longest median perfusion time again presumably reflecting that these livers were from patients with more advanced liver cirrhosis. Moreover, human hepatocytes isolated from ALD demonstrate significantly less albumin production when compared to the other human hepatocytes supporting the hypothesis that prior alcohol exposure may affect the function of these hepatocytes. Our data further highlight that tissue from patients with biliary cirrhosis (PBC or PSC) provides high yields of hepatocytes (success rate 71%). Furthermore, such hepatocytes demonstrate the highest levels of albumin and urea production. As discussed below, this success rate was similar to that we obtained using non-diseased liver tissue. In biliary cirrhosis cytoprotective agents such as ursodeoxycholic acid (UDCA) are routinely used therapeutically. The ability of UDCA to inhibit the accumulation of reactive oxygen species (ROS) [15] and its known anti-apoptotic effects may provide hepato-protection [16]. Indeed in our reported series all patients with biliary cirrhosis were taking UDCA at the time of liver transplantation. Thus UDCA may promote human hepatocytes isolation in an analogous way to that suggested for the antioxidant, N-acetylcysteine (NAC) [3]. Conversely, alcohol consumption increases ROS production which instigates hepatocyte injury during ALD [17] as well as inducing toxic metabolites and cytokines that perpetuate hepatocyte injury. It is thus possible that hepatocytes from patients with ALD are sensitised to the cellular stress induced by enzymes used in the digestion process resulting in increased hepatocyte cell death and lower cell viability and yields. The yields from end-stage cirrhotic livers other than ALD and biliary cirrhosis were very poor results (success rate 13%) and we would not recommend the routine use of such livers for cell isolation.

We report a success rate of 53% for hepatocyte isolation from normal resected livers, which is lower than that reported by other authors [7], [18]. There are several potential explanations for this. Other studies used different enzyme cocktails and may have been more selective when deciding which livers to process [1]. Recent studies have used the enzyme liberase to isolate hepatocytes from normal resected liver tissue and have reported higher cell viabilities and cell yields [1]. We concur with previous authors that normal resected tissue is a good source of human hepatocytes. However, our recent study suggests that human hepatocytes isolated from normal resected liver tissue may differ functionally in their response to physiological stimuli [4]. Most authors have previously assumed that the function of human hepatocytes isolated from normal liver tissue was consistent between different sources [18]. In light of our findings further functional analyses of human hepatocytes isolated from different liver diseases should be carried out before conclusions can be drawn about their ‘normality’.

The isolations we carried out using liver wedges from benign resections and cut down specimens gave good success rates (64%) compared to donor liver tissue (29%) consistent with results reported by others [7]. The donor livers used in our study had been turned down for transplantation as a result of macrosteatosis and as Figure 2b demonstrates human hepatocytes from these steatotic livers also exhibit micro-vesicular steatosis. Within the steatotic livers the rate of successful hepatocyte isolation was greater than in those with mild compared with moderate or severe steatosis (70% vs. 20%). Previous authors have also noted that high degrees of steatosis generally did not favour successful cell isolation [6], [7]. Steatotic hepatocytes have higher intracellular ROS levels and are thus potentially more susceptible to damage [3]. The recent work of Sagias *et al* suggests that the addition of NAC to perfusion fluids may improve hepatocyte yields from steatotic livers. Another, contributory factor for the lower viability and yield of hepatocytes from donor liver is the longer cold ischaemic time that donor livers have been exposed to when compared to normal livers (Table 1).

Previous authors have suggested that the optimal liver wedge weight for human hepatocyte isolation is approximately 100 g. We agree with this and in the present study the majority of liver wedges were between 80–120 g. In our study a median perfusion time of 2.5 min was required to digest the livers, as judged by the admission of a digit into the liver substance. Other studies report digestion times ranging from 20–47 min (1) [19]. This could simply be a reflection of different enzyme types, concentrations or differences between batches. In our series the same enzyme batches were used for all isolations. The enzyme cocktail we used allowed isolation of cells from end-stage cirrhotic livers and it is possible that this enzyme concentration may be excessive for normal resected and donor liver tissue thereby contributing to our low yield and viability compared with other reports.

Although perfusion time was significant in the univariable analysis it fell out during multivariable analysis. The reason for this is likely to be correlation with the time delay between hepatectomy and beginning of liver perfusion (Spearman's Rho  = .377, p<.001) which remained significant even after adjusting for perfusion time.

Thus we provide evidence to suggest that the time delay between hepatectomy and beginning of liver perfusion should be kept to less than 3 hours. From our experience of isolating human hepatocytes from cirrhotic livers after an initial learning curve the operator becomes skilled at recognising when adequate digestion has occurred thereby avoiding overdigestion of tissue.

In conclusion, we report that the time delay between hepatectomy and beginning of liver perfusion is the most important factor in determining the likelihood of success of the procedure. Furthermore, we suggest the shortest possible digestion time is desirable. If protocols similar to ours are followed human hepatocytes can be isolated successfully from normal and diseased liver tissue.
